# Bisphenol A‐Induced Vascular Endothelial Dysfunction: Protective Effects of *Nigella sativa* Oil and Thymoquinone

**DOI:** 10.1002/fsn3.71290

**Published:** 2025-11-28

**Authors:** Masoumeh Fadishei, Mahdieh Sadat Mohsenzadeh, Bibi Marjan Razavi, Mohsen Imenshahidi, Seyed Ahmad Mohajeri, Mahboubeh Ghasemzadeh Rahbardar, Hossein Hosseinzadeh

**Affiliations:** ^1^ Department of Pharmacodynamics and Toxicology, School of Pharmacy Mashhad University of Medical Sciences Mashhad Iran; ^2^ Food Control Laboratory, Department of Food and Drug Mashhad University of Medical Sciences Mashhad Iran; ^3^ Department of Pharmacodynamics and Toxicology, School of Pharmacy, Targeted Drug Delivery Research Center Mashhad University of Medical Sciences Mashhad Iran; ^4^ Pharmaceutical Research Center, Pharmaceutical Technology Institute Mashhad University of Medical Sciences Mashhad Iran

**Keywords:** bisphenol A, endothelial dysfunction, HUVECs, isolated rat aorta, *Nigella sativa* oil, thymoquinone

## Abstract

In the present study, the effectiveness of *Nigella sativa* oil (NSO) and thymoquinone (TQ), a major ingredient in *Nigella sativa*, on vascular endothelial dysfunction induced by bisphenol A (BPA) was assessed. Male Wistar rats were exposed to NSO (84 μL/kg), TQ (2 mg/kg), BPA (10 mg/kg), BPA plus NSO (21, 42, or 84 μL/kg), or BPA plus TQ (0.5, 1, or 2 mg/kg) (*n* = 6 per group). After the treatment period, oxidative and nitrosative stress markers in both aorta and human umbilical vein endothelial cells (HUVECs) were evaluated. In HUVECs, protein levels of phospho‐endothelial nitric oxide synthase (p‐eNOS) and adhesion molecules (vascular cell adhesion molecule‐1 [VCAM‐1] and E‐selectin) were measured. In addition, contractile responses to potassium chloride (KCl) and phenylephrine (PE) and relaxant responses to acetylcholine (ACh) and sodium nitroprusside (SNP) were evaluated in the rat aorta. BPA caused a significant increase in aorta lipid peroxidation, elevated reactive oxygen species (ROS), nitric oxide (NO), VCAM‐1, and p‐eNOS in HUVECs, and decreased aorta responses to KCl and PE. These changes were ameliorated by NSO and TQ administration. Furthermore, NSO and TQ alone increased the vasorelaxation responses induced by ACh and SNP. These findings suggest that NSO and TQ can protect the vascular endothelium against BPA‐induced damage, probably resulting from their antioxidant activity, as well as their ability to attenuate cell adhesion molecules.

## Graphical abstract

BPA exposure in rats and HUVECs impaired aortic contractility, increased lipid peroxidation, ROS, NO, VCAM‑1, and p‑eNOS, and blunted relaxant responses. Co‑treatment with NSO or TQ restored KCl/PE‑induced contractions, enhanced ACh‑ and SNP‑mediated vasorelaxation, and lowered oxidative/nitrosative stress and adhesion molecule expression. These results indicate NSO and TQ protect vascular endothelium from BPA‑induced dysfunction via antioxidant and anti‑adhesion mechanisms.

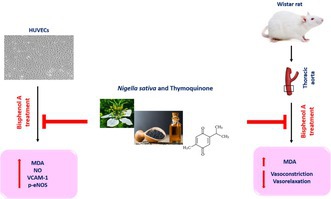

**Abbreviations**
2‐ME: 2‐mercaptoethanol
ACh: acetylcholine
BPA: bisphenol A
DMEM‐F12: Dulbecco's modified Eagle's medium‐F12
DMSO: dimethyl sulfoxide
EDTA: ethylenediaminetetraacetic acid
EGTA: ethylene glycol tetraacetic acid
eNOS: endothelial nitric oxide synthase
eNOS: endothelial nitric oxide synthase
FBS: fetal bovine serum
HPLC: high‐pressure liquid chromatography
HUVECs: human umbilical vein endothelial cells
ICAM: intercellular adhesion molecule
KHBS: Krebs–Henseleit buffer solution
MDA: malondialdehyde
MTT: 3‐(4,5‐dimethyl‐2‐thiazolyl)‐2,5‐diphenyl‐2H‐tetrazolium bromide
NO: nitric oxide
NSO:
*Nigella sativa* oil
PE: phenylephrine
PMSF: phenylmethanesulfonyl fluoride
PVDF: polyvinylidene difluoride
ROS: reactive oxygen species
SDS: sodium dodecyl sulfate
SEM: standard error of the mean
SNP: sodium nitroprusside
TQ: thymoquinone
VCAM‐1: vascular cell adhesion molecule‐1

## 1 Introduction

Bisphenol A (BPA) is a plastic monomer commonly used in the synthesis of epoxy resins and polycarbonate (Mohsenzadeh, Razavi, Imenshahidi, Tabatabaee Yazdi, et al. 2021). It has been known that BPA can be released from polycarbonate plastics, plastic coatings, thermal papers, and metal waste into the environment, food, and water (Mikołajewska et al. 2015). The main route of human exposure to this chemical is reported through food (Corrales et al. 2015). A previous study has shown that the higher level of BPA in human urine is related to various cardiovascular disorders including hypertension, heart attack, and angina (Gao and Wang 2014). Moreover, BPA chronic and subchronic exposure in rodents increased the risk of hypertension and dyslipidemia and also induced susceptibility to atherosclerosis (Fang et al. 2014). The results of in vitro studies showed that BPA exhibited high levels of ROS, adhesion molecules and p‐eNOS (ser 1177) expression, inflammatory responses, and proangiogenic effects in human umbilical vein endothelial cells (HUVECs). Endothelial dysfunction is considered an early and critical event in the pathogenesis of atherosclerosis, as factors that cause dysfunction lead to endothelial damage and initiation of the atherogenic process (Andersson and Brittebo 2012; Fang et al. 2015).

Atherosclerosis is defined as an inflammatory arterial disease characterized by endothelial dysfunction. Vascular endothelial dysfunction is associated with impaired vasoreactivity, deficiency in endothelial nitric oxide synthase (eNOS) and nitric oxide (NO) function, adhesion molecule expression, and reactive oxygen species (ROS) generation (Abbasnezhad et al. 2019).

Overproduction of ROS and oxidative stress has been reported to induce vascular endothelial dysfunction in human and animal models (Andersson and Brittebo 2012; Wang et al. 2006). ROS can interact and inactivate NO. Therefore, vascular oxidative stress can decrease the bioavailability of NO. NO is known as the main factor in regulating vascular tonicity and permeability. In addition, NO initiates relaxation in vascular smooth muscle cells. Reduced bioavailability of NO results in impaired vascular relaxation, endothelial dysfunction, and increased risk of atherosclerosis (Ogita and Liao 2004). Endothelial NOS serves as a key enzyme in the production of NO. Under oxidative stress conditions, eNOS becomes dysfunctional and produces superoxide rather than NO, thereby impairing endothelium‐dependent vasorelaxation (Kawashima and Yokoyama 2004). Another major component of vascular endothelial dysfunction is surface adhesion molecules (vascular cell adhesion molecule‐1 [VCAM‐1] and E‐selectin). Activated vascular endothelium expresses adhesion molecules on leukocytes and endothelium to facilitate leukocyte migration across the endothelial cells (Galkina and Ley 2007).

In the last few years, the role of medicinal plants and their bioactive ingredients as a new source of therapeutic agents in preventing and treating cardiovascular disorders has been widely investigated (Adegbola et al. 2017; Al‐Snafi 2017; Michel et al. 2020; Mota 2016; Rouhi‐Boroujeni et al. 2017). *Nigella sativa* , black seed or black cumin, is a medicinal plant from the family of Ranunculaceae. The health‐beneficial effects of *N. sativa* mostly reside in the seeds. Alkaloids, proteins, essential oils, flavonoids, polyphenols, and thymoquinone (TQ) have been identified in *N. sativa* seeds (Hosseini et al. 2017). Thymoquinone is the most active and abundant ingredient of the volatile oil extracted from the black seed, *N. sativa* (Tavakkoli et al. 2017). *N. sativa* and TQ have been reported to be used as protective and therapeutic agents against hypertension, diabetes, inflammation, bronchitis, and influenza (Ali and Blunden 2003; Entok et al. 2014). *N. sativa* and TQ are also reported to be effective against aflatoxicosis (Ates et al. 2022).

In previous studies, it was found that *N. sativa* oil (NSO) and its active ingredient TQ have cardiovascular beneficial effects by reducing the ROS level (Oskouei et al. 2018; Razavi and Hosseinzadeh 2014), maintaining the activity of antioxidant enzymes (Danaei et al. 2019; Oskouei et al. 2018; Randhawa et al. 2013; Razavi and Hosseinzadeh 2014), decreasing inflammatory cytokine levels (Ojha et al. 2015), increasing plasma NO levels (Razavi and Hosseinzadeh 2014), regulating autophagy and apoptosis (Xiao et al. 2018), improving histopathological changes (Rahmani and Aly 2015; Randhawa et al. 2013), and also blocking calcium channels (Razavi and Hosseinzadeh 2014).

Pathological disorder of the endothelial cells has a key role in the pathogenesis of cardiovascular diseases (Vanhoutte et al. 2009). On the other hand, the positive effect of *N. sativa* and TQ on the vascular endothelium and overall vascular function has attracted much attention. For example, TQ could protect endothelial dysfunction induced by pyrogallol in the aortic tissue of rabbits, at least in part, through its antioxidant capacity and enhancement of NO bioavailability (El‐Agamy and Nader 2012). To the best of our knowledge, there is little published evidence demonstrating the beneficial effects of *N. sativa* and its active ingredient TQ on attenuating vascular endothelial dysfunction induced by BPA. Hence, our study was designed to investigate the impact of *N. sativa* oil and TQ on protecting BPA‐induced endothelial dysfunction in rat aorta and HUVECs, and also evaluate possible underlying mechanism(s). Towards this end, constrictive responses to potassium chloride (KCl) and phenylephrine (PE) and vasorelaxation responses to acetylcholine (ACh) and sodium nitroprusside (SNP) were evaluated in isolated rat aortic rings. Moreover, oxidative and nitrosative stress markers in aorta and HUVECs, as well as protein levels of p‐eNOS and adhesion molecules (VCAM‐1 and E‐selectin) in HUVECs were evaluated. In our previous study, the beneficial effects of NSO and TQ on alleviating BPA‐induced metabolic disorders, including lipid profile, liver biomarkers, serum adipokines, inflammatory cytokines, fasting blood sugar, serum insulin level, and hepatic protein levels of IRS‐1, PI3K, and Akt have been reported (Fadishei et al. 2021). It should be mentioned that our previous research was conducted on a separate group of animals and focused on metabolic outcomes, while the present study independently investigates vascular endothelial dysfunction.

## 2 Materials and Methods

### 2.1 Chemicals and Materials

BPA (Sigma, USA), thymoquinone (TQ; Sigma, Germany), sodium orthovanadate (Na_3_VO_4_; Sigma, India), sodium deoxycholate (Sigma, New Zealand), phenylephrine (PE; Sina Darou, Iran), acetylcholine (ACh; Sigma, Switzerland), sodium nitroprusside (SNP; Rotapharma, Spain), NaF (Sigma, USA), dry skim milk (Quet Lab, UK), fetal bovine serum (FBS; Gibco, USA), Dulbecco's modified Eagle's medium‐F12 (DMEM‐F12; Caisson, USA), and trypsin (Caisson, USA).

Phenylmethanesulfonyl fluoride (PMSF), ethylenediaminetetraacetic acid (EDTA), ethylene glycol tetraacetic acid (EGTA), β‐glycerol phosphate, 3‐(4,5‐dimethyl‐2‐thiazolyl)‐2,5‐diphenyl‐2H‐tetrazolium bromide (MTT), and penicillin/streptomycin were purchased from Sigma‐Aldrich (Germany). Bromophenol blue, Tris, 2‐mercaptoethanol (2‐ME), sodium dodecyl sulfate (SDS), glycerol, and dimethyl sulfoxide (DMSO) were from Merck (Germany).

Protease and phosphatase inhibitor cocktail and Pierce ECL (Thermo Fisher Scientific, USA), protein assay kit (Bradford reagent; Bio‐Rad, USA), polyvinylidene difluoride (PVDF; Bio‐Rad, USA). Mouse monoclonal antibody against VCAM‐1 (Santa Cruz Biotechnology, USA), rabbit polyclonal antibodies against E‐selectin and phospho‐eNOS (ser1177) (Santa Cruz Biotechnology, USA), anti‐rabbit or anti‐mouse IgG, HRP‐linked antibodies and β‐actin (Cell Signaling Technology, USA) were used in the present study.

*N. sativa* seeds were obtained from Esfahan, Iran. Botanical identification of the seeds was done by the School of Traditional Medicine, Mashhad, Iran. Cold‐pressed oil was prepared by Dr. Yousefi, MD, Ph.D (School of Traditional Medicine, Mashhad University of Medical Sciences, Iran) as follows: 10 g of dry and powdered seeds were weighed. The pressing process was carried out at a temperature below 35°C in chrome‐nickel cold‐pressed oil machines (Household Oil Press, Oily YD‐ZY‐03A, Germany) without any chemical or heating process. The oil was filtered through 100% cotton filters with a thickness of 2 mm to separate solid particles. To ensure sterility and safety for intraperitoneal (IP) administration, the extracted oil was passed through a 0.22 μm sterile syringe filter under aseptic conditions before storage. The sterilized oil was then poured into sterile glass bottles with nitrogen gas and stored at −20°C until analysis. The efficiency was up to 15%.

### 2.2 Animals and Treatment

A total of 88 adult male Wistar rats, weighing 210–260 g, were purchased from the Animal Center of Pharmacy School, Mashhad, Iran. The animals were adapted under a 12–12 h light–dark cycle, at 25°C ± 2°C, and 50% ± 10% humidity in the plastic cages, with six rats per cage. All rats had free access to water and food. The experimental procedures applied in the present study were approved by the Ethical Committee at Mashhad University of Medical Sciences (941399; 2016). Rats were treated based on the following protocol: (1) TQ control: BPA vehicle (tragacanth gel 1%, gavage) and TQ vehicle (tween 80/oleic acid, 1:3, 1%, IP). (2) NSO control: BPA vehicle (tragacanth gel 1%, gavage) and NSO vehicle (tween 80, 3%, IP). (3) TQ (2 mg/kg, IP). (4) NSO (84 μL/kg, IP). (5) BPA (10 mg/kg, gavage). (6, 7, and 8) BPA (10 mg/kg, gavage) + NSO (21, 42, and 84 μL/kg, IP, separately). (9, 10, and 11) BPA (10 mg/kg, gavage) + TQ (0.5, 1, and 2 mg/kg, IP, separately).

BPA was suspended in 1% tragacanth gel. NSO was emulsified in distilled water using 3% Tween 80 with gentle shaking. TQ was dissolved in Tween 80/oleic acid (1:3) and then emulsified with distilled water to obtain a 1% solution. All treatments were administered at a volume of approximately 1 mL/kg body weight. According to previous studies, chronic treatment with tween/water vehicles showed no adverse effects in rats (Gad et al. 2006; Turner et al. 2011). Control groups received the corresponding vehicles (without active ingredients) in equivalent volumes. The solutions were freshly prepared each day before administration. Rats were treated daily for 54 days. During the treatment period, no mortality or significant adverse effects were observed, and all groups were completed with the initial number of animals. TQ and NSO were given 30 min after BPA administration. According to a previous study, after oral administration of BPA (400 μg/kg), the maximum plasma concentration of BPA was reached 20 min after oral administration (Draganov et al. 2015). Therefore, in the current work, TQ and NSO were administered after 30 min of BPA gavage when BPA plasma concentration reached its maximum. The dose selection and administration route for these compounds were according to a previous part of our study (Fadishei et al. 2021). After 54 days of treatment, animals were killed by decapitation. Some aortas were removed, washed with cold normal saline, and stored at −80°C until analysis. The other aortic tissues were collected and placed in isolated aorta apparatus. Aortic rings (4–5 mm) were attached in an organ bath system (AD Instruments, Australia) between two stainless steel hooks, under a resting tension of 2 g, and connected to a force transducer for recording isometric tension (PowerLab; AD Instruments, Australia).

### 2.3 Determination of Lipid Peroxidation Marker

The main marker of oxidative stress, malondialdehyde (MDA), was measured as follows (Ohkawa et al. 1979). Briefly, aortic tissue was homogenized with KCl solution (1.15%) for the preparation of a tissue homogenate of 10%. Three milliliters of 1% phosphoric acid and 1 mL of 0.6% TBA were added to 0.5 mL of 10% aortic homogenate. The mixture was boiled for 45 min. Afterward, 4 mL of *n*‐butanol was added and vortexed for 1 min. The upper layer (butanol phase) was separated by centrifugation at 3000 *g* for 10 min and the absorbance was determined at 532 nm. The MDA content was expressed as nmol/g tissue.

### 2.4 Cell Culture

HUVEC cells, obtained from Pasteur Institute (Tehran, Iran), were cultured in a DMEM‐F12 medium with 10% FBS, penicillin (100 U/mL), and streptomycin (100 mg/mL). Cells were incubated in a humidified atmosphere of 5% CO_2_ at 37°C and selected for the experiments at the confluence of 80% in culture flasks.

#### 2.4.1 MTT Assay

Cell viability was evaluated by the methyl thiazol tetrazolium bromide (MTT) assay. Cells were seeded in a 96‐well cell culture plate at a density of 10^4^ cells per well. BPA IC_50_ value and non‐toxic concentrations of TQ and NSO were calculated by the MTT assay. For BPA, various concentrations ranging from 25 to 800 μM were added and the plate was incubated for 24 h. In other plates, adhered cells were treated with different concentrations of TQ (3.125–100 μM) and NSO (0.039–10 μL/mL) for 48 h. To assess the protective effects of TQ/NSO on the BPA IC_50_ value, HUVECs were pretreated with TQ (3.125, 6.25, and 12.5 μM) or NSO (0.078, 0.156, and 0.31 μL/mL) for 24 h and then BPA at the IC_50_ value was added to each well. After 24 h, 0.5 mg/mL of MTT solution was added to each well and incubated for 3 h at 37°C. Afterward, the medium was removed, dimethylsulfoxide (DMSO; 100 μL) was added and shaken up to 20 min for solving formazan crystals. Absorbance was measured at 545 nm (630 nm as a reference) by an ELISA reader (Start Fax‐2100, UK) (Hosseinzadeh et al. 2008).

#### 2.4.2 Determination of Intracellular NO

The Griess reaction method was used to measure the released NO in the cell culture medium. Cells were seeded in a 96‐well culture plate (1 × 10^4^ cells/well), pretreated with three different concentrations of TQ (3.125, 6.25, and 12.5 μM) or NSO (0.078, 0.156, and 0.31 μL/mL) for 24 h, then subjected to BPA toxicity (at IC_50_ concentration) for 24 h. Fifty microliters of cell culture supernatant was mixed with 50 μL Griess reagent (sulfanilic acid and 2‐naphthylamine in phosphoric acid). The absorbance of the pink color developed after 30 min was measured with an ELISA plate reader (Thermo Electron) at a wavelength of 540 nm (Zhang et al. 2014).

#### 2.4.3 Determination of Intracellular ROS

ROS production was evaluated using the 2′, 7′‐dichlorofluorescein diacetate (DCFH‐DA) method. DCFH‐DA oxidation in the presence of intracellular ROS causes fluorescent dichlorofluorescein (DCF) (Tong et al. 2016). HUVECs were pretreated with TQ (3.125, 6.25, and 12.5 μM) or NSO (0.078, 0.156, and 0.31 μL/mL) followed by exposure to BPA (IC_50_ value) as previously described. Then, cells were washed with PBS and incubated with 10 μM DCFH‐DA. After 30 min, the intensity of fluorescence at the excitation/emission wavelength of 485/527 nm was recorded by a microplate reader (Thermo Electron).

#### 2.4.4 Western Blot

In HUVECs, the protein levels of VCAM‐1, E‐selectin, and p‐eNOS (ser1177) were evaluated by western blot. First, the cells were lysed in cold lysis buffer (50 mM Tris; pH 7.4, 2 mM EDTA, 2 mM EGTA, 10 mM NaF, 1 mM Na_3_NO_4_, 10 mM β‐glycerol phosphate, 0.2% w/v sodium deoxycholate, 10% v/v 2‐ME, 1 mM PMSF, and complete protease and phosphatase inhibitor cocktail). The Bradford assay was used to estimate the concentration of protein (Bradford 1976). Afterward, the supernatant (extracted proteins) was mixed with loading buffer, containing 100 mM Tris‐base, 10% v/v 2‐ME, 20% w/v SDS, 20% v/v glycerol, and 0.2% w/v bromophenol blue, heated for 5 min, and frozen at −80°C.

Total proteins were separated by 10% SDS‐PAGE and wet‐transferred to PVDF membranes. After blocking with 5% dry skim milk (for VCAM‐1 and E‐selectin) or 5% bovine serum albumin (for p‐eNOS; ser 1177), at RT for 2 h, the membranes were incubated with the appropriate primary antibodies (1:1000 dilution) overnight. Membranes were washed with Tris‐buffered saline Tween 20 (TBST) and probed with a 1:3000 dilution of HRP anti‐rabbit or anti‐mouse IgG at RT for 90 min. Finally, protein band detection was done by enhanced chemiluminescence (Pierce ECL Western Blotting Substrate) and Alliance 4.7 Gel doc (UK). The intensity of the protein bands was normalized against β‐actin.

### 2.5 Isolated Aorta Studies

#### 2.5.1 Tissue Preparation

Adult rats were decapitated to obtain the isolated aorta. After thoracotomy, aortic tissue was rapidly excised, and placed into cold Krebs buffer with the composition of 118 mM NaCl, 4.7 mM KCl, 1.2 mM NaH_2_PO_4_, 1.2 mM MgSO_4_, 25 mM NaHCO_3_, 11.1 mM glucose, and 2.5 mM CaCl_2_, and cleaned from fat and connective tissue. Aortic rings (4–5 mm long) were mounted between two stainless steel wires in an organ bath (AD Instrument, Australia) under a resting tension of 2 g and equilibrated for 45 min in Krebs solution (pH 7.38–7.5) at 37°C and the gas mixture of 95% O_2_ and 5% CO_2_ (Rameshrad et al. 2016). Tension was recorded by a transducer and a computer‐assisted data acquisition system (PowerLab, AD Instrument, Australia).

#### 2.5.2 Determination of Aorta Viability, Contractility, and Endothelium Integrity

To confirm tissue viability and obtain maximum tension, the contractile response of segments to cumulatively increasing concentrations of KCl (20–80 mM) and PE (10^−9^–10^−4^ M) was measured, respectively. To establish the integrity of endothelial cells, the vasorelaxant response to cumulatively increasing concentrations of ACh (10^−9^–10^−4^ M) in pre‐exposed rings with PE (10^−6^ M) was measured. Besides, endothelium‐independent relaxation to cumulatively increasing concentrations of SNP (10^−12^–10^−8^ M) in pre‐exposed rings with PE (10^−6^ M) was recorded (Rameshrad et al. 2018). The effect of TQ, NSO, and BPA on the pD_2_ (−log EC_50_) value of ACh in rat aorta after 54 days of treatment was also evaluated.

### 2.6 Statistical Analysis

All data were expressed as mean ± SEM. To compare the means, a one‐way analysis of variance (ANOVA) followed by the Tukey–Kramer test was used. Two‐way ANOVA and the Tukey–Kramer test were used to evaluate any differences between the concentration‐dependent response curves. IC_50_ and pD_2_ values were calculated by non‐linear regression analysis. *p* values less than 0.05 were considered significant. Statistical analysis was performed by Prism GraphPad Software (La Jolla, California, USA).

## 3 Results

### 3.1 Determination of Aortic Lipid Peroxidation

**FIGURE 1:**
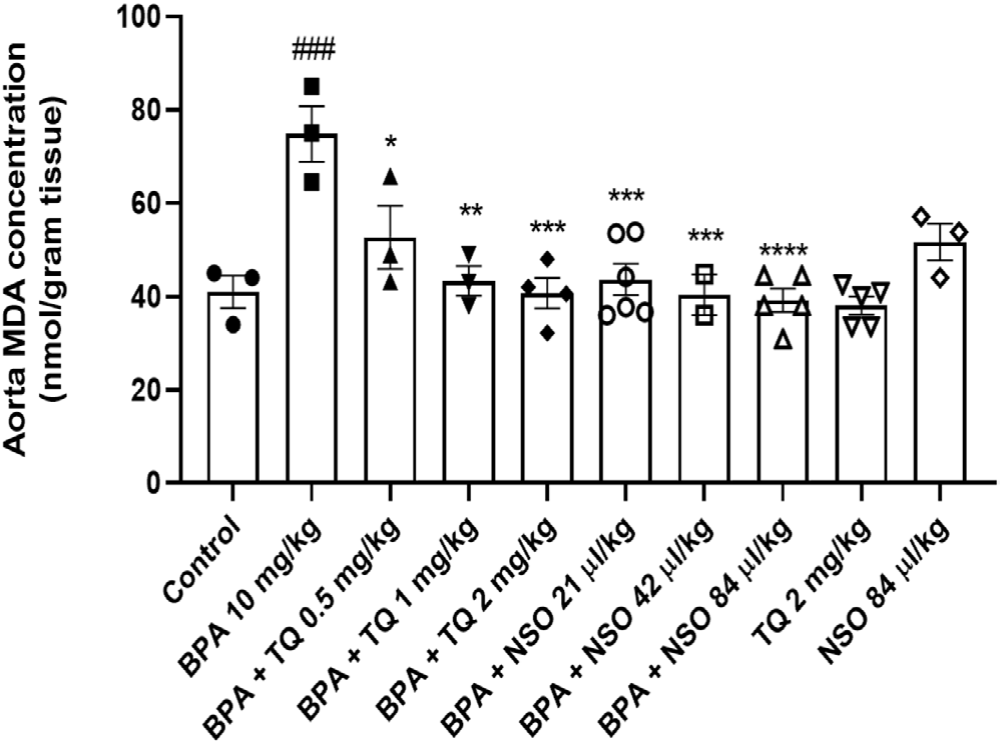
Dot plots/box plots of BPA, TQ, and NSO effects on aortic MDA levels. Male Wistar rats were treated with BPA (10 mg/kg, gavage) and/or TQ (0.5, 1, and 2 mg/kg, IP) or NSO (21, 42, and 84 μL/kg, IP) for 54 days. ^###^ *p* < 0.001 compared to the control group; ****p < 0.0001, ***p < 0.001, **p < 0.01, and *p < 0.05 compared to the BPA group. BPA, bisphenol A; MDA, malondialdehyde; NSO, *Nigella sativa* oil; TQ, thymoquinone.

In Figure 1, BPA exposure increased the aorta MDA level in comparison with the control (*p* < 0.001). Administration of TQ at 0.5 (*p* < 0.05), 1 (*p* < 0.01), and 2 mg/kg (*p* < 0.001) with BPA decreased MDA level compared to the BPA group. Besides, after concurrent administration of NSO at doses of 21 (*p* < 0.001), 42 (*p* < 0.001), and 84 μL/kg (*p* < 0.0001) with BPA, MDA level was profoundly attenuated compared to BPA alone.

### 3.2 Effects of TQ, NSO, and BPA on Cytotoxicity in HUVECs

**FIGURE 2:**
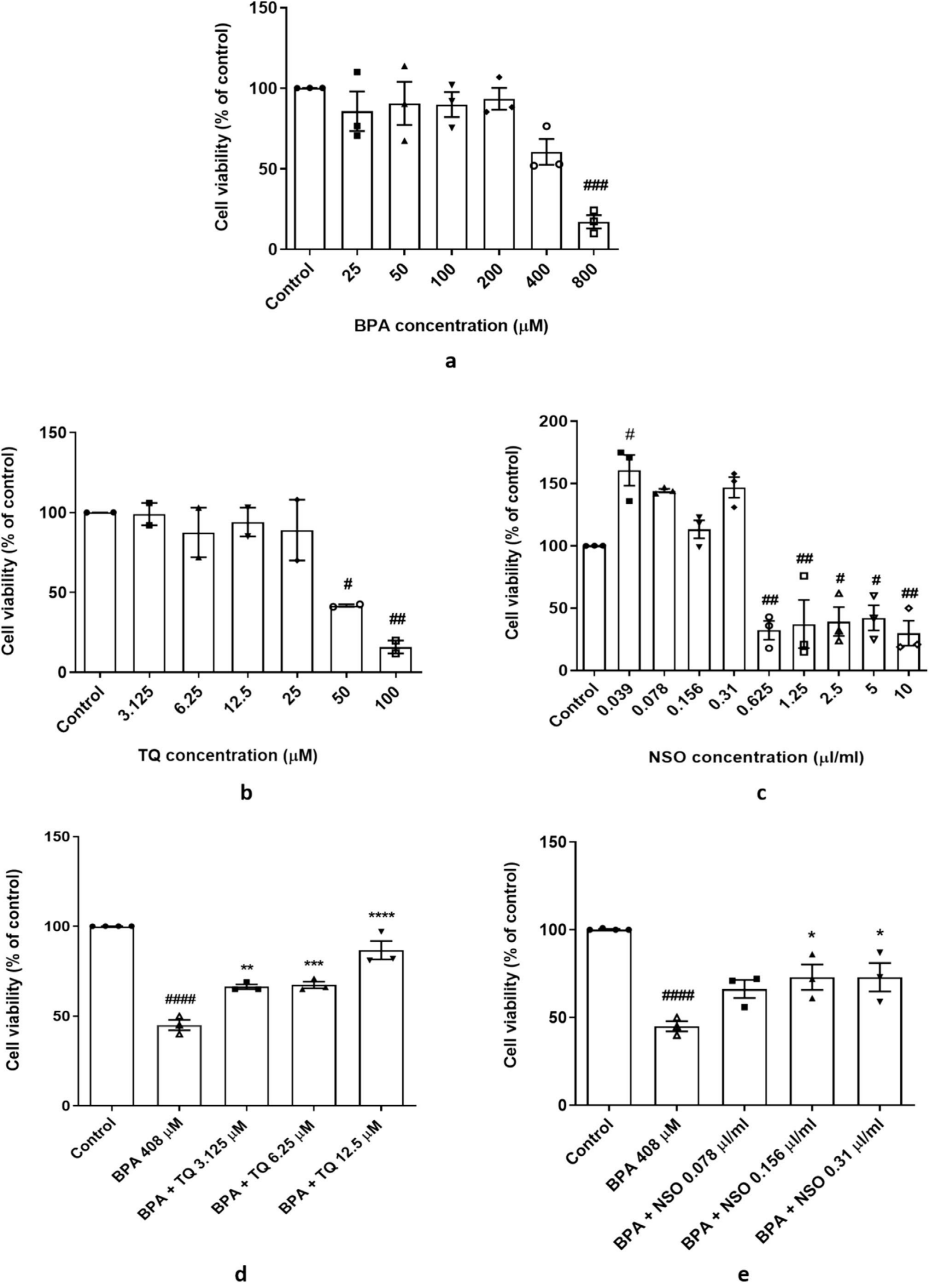
Dot plots/box plots of effects of BPA (a), TQ and NSO alone (b, c), and TQ or NSO pretreatment with BPA (d, e) on HUVECs viability by MTT assay. Cells were treated with BPA (25, 50, 100, 200, 400, and 800 μM), TQ (3.125, 6.25, 12.5, 25, 50, and 100 μM), and NSO (0.039, 0.078, 0.156, 0.31, 0.625, 1.25, 2.5, 5, and 10 μL/mL). ^####^ *p* < 0.0001, ^###^ *p* < 0.001, ^##^ *p* < 0.01, and ^#^ *p* < 0.05 compared to the control group; ****p < 0.0001, ***p < 0.001, **p < 0.01, and *p < 0.05 compared to the BPA group. BPA, bisphenol A; HUVECs, human umbilical vein endothelial cells; NSO, *Nigella sativa* oil; TQ, thymoquinone.

The IC_50_ of BPA was 408 μM, and TQ at concentrations of 3.125–25 μM or NSO at 0.039–0.31 μL/mL was not cytotoxic (Figure 2a–c). Pretreatment of HUVECs with TQ 3.125 (*p* < 0.01), 6.25 (*p* < 0.001), and 12.5 μM (*p* < 0.0001; Figure 2d) or NSO 0.156 and 0.31 μL/mL (*p* < 0.05; Figure 2e) could significantly attenuate the cytotoxicity induced by 408 μM of BPA.

### 3.3 Effects of TQ, NSO, and BPA on NO and ROS Levels in HUVECs

**FIGURE 3:**
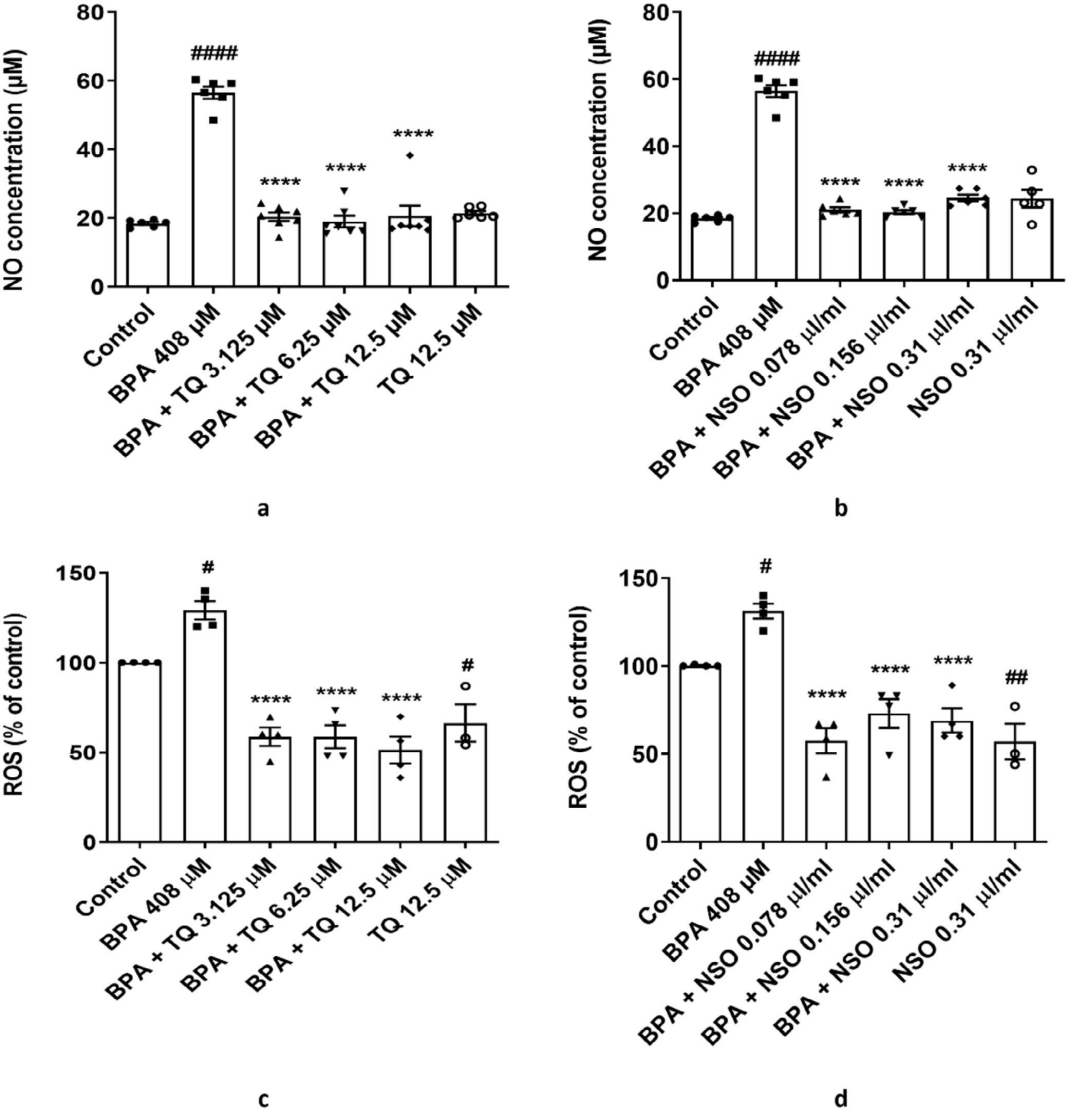
Dot plots/box plots of TQ (a, c) and NSO (b, d) effects on BPA‐induced NO and ROS generation in HUVECs. Cells were pretreated with TQ (3.125, 6.25, and 12.5 μM) or NSO (0.078, 0.156, and 0.31 μL/mL) and then exposed to BPA (408 μM) for 24 h. ^####^ *p* < 0.0001, ^##^ *p* < 0.01, and ^#^ *p* < 0.5 compared to the control group; ****p < 0.0001 compared to the BPA group. BPA, bisphenol A; HUVECs, human umbilical vein endothelial cells; NO, nitric oxide; NSO, *Nigella sativa* oil; TQ, thymoquinone.

BPA exposure (408 μM) significantly increased NO content (*p* < 0.0001; Figure 3a,b) and reactive oxygen species (ROS) formation (*p* < 0.05; Figure 3c,d) compared to the control. Pretreatment with TQ (Figure 3a,c) or NSO (Figure 3b,d) at three concentrations markedly attenuated BPA‐induced NO and ROS generation in comparison with the BPA group (*p* < 0.0001). By treatment with TQ or NSO alone, ROS levels were significantly reduced (*p* < 0.05 in Figure 3c, *p* < 0.01 in Figure 3d vs. control group).

### 3.4 Effects of TQ, NSO, and BPA on the Protein Levels of p‐eNOS (ser 1177), VCAM‐1, and E‐Selectin in HUVECs

**FIGURE 4:**
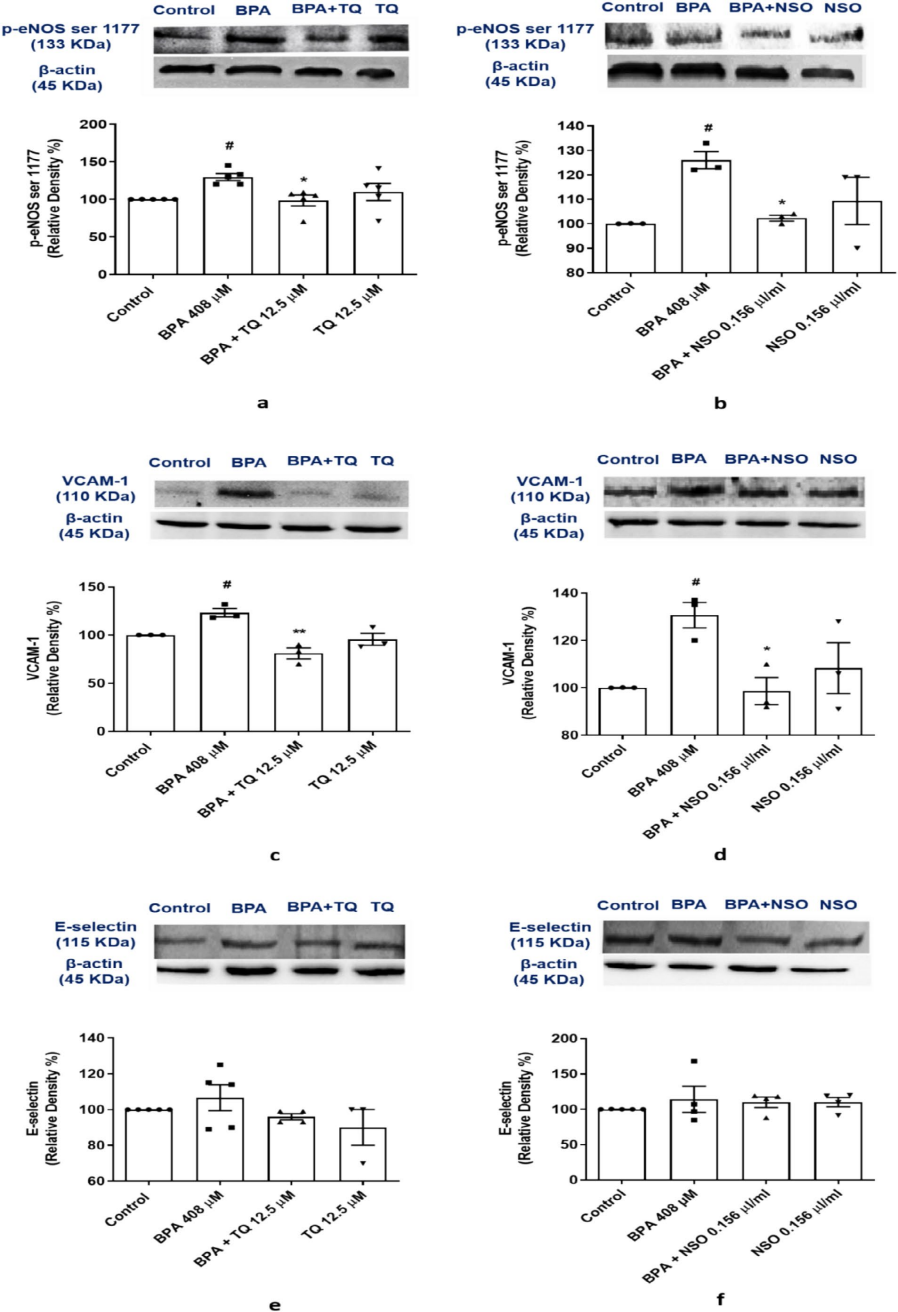
Dot plots/box plots of BPA, TQ, and NSO effects on protein expression of p‐eNOS [ser 1177 (a, b)], VCAM‐1 (c, d), and E‐selectin (e, f) in HUVECs. Western blot analysis was performed to determine the protein levels in HUVECs exposed to TQ (12.5 μM) or NSO (0.156 μL/mL) 24 h before the co‐treatment with BPA (408 μM, 24 h). ^#^ *p* < 0.05 compared to the control group; **p < 0.01 and *p < 0.05 compared to the BPA group. BPA, bisphenol A; eNOS, endothelial nitric oxide synthase; HUVECs, human umbilical vein endothelial cells; NSO, *Nigella sativa* oil; TQ, thymoquinone; VCAM‐1, vascular cell adhesion molecule‐1.

Our results showed that BPA treatment significantly up‐regulated the protein expression of p‐eNOS (*p* < 0.05; Figure 4a,b) and VCAM‐1 (*p* < 0.05; Figure 4c,d) relative to rats in the control group. Besides, the protein level of E‐selectin in the BPA group was increased non‐significantly as compared to the control (Figure 4e,f). Pretreatment of HUVECs with TQ at 12.5 μM decreased the protein level of p‐eNOS (*p* < 0.05; Figure 4a) and VCAM‐1 (*p* < 0.01; Figure 4c). Moreover, pretreatment with NSO at 0.156 μL/mL decreased the protein level of p‐eNOS (*p* < 0.05; Figure 4b) and VCAM‐1 (*p* < 0.05; Figure 4d) in comparison with the BPA‐treated cells.

### 3.5 Aorta Vasoconstrictive Responses to KCl and PE

**FIGURE 5:**
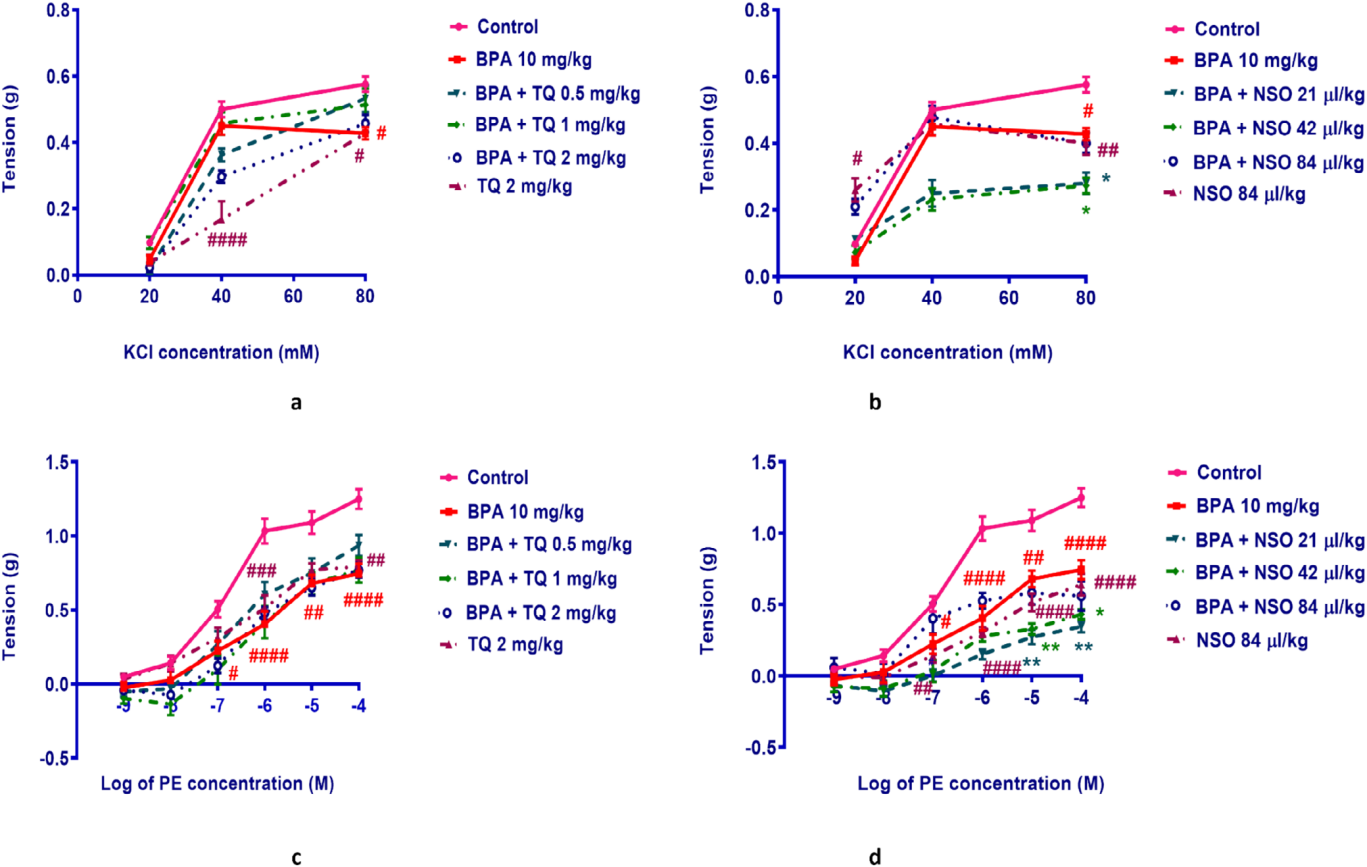
Concentration–response curve presenting the vasotonic responses of rat aorta rings to the cumulative concentrations of KCl (a, b) and PE (c, d). Male Wistar rats were treated with BPA (10 mg/kg, gavage) and/or TQ (0.5, 1, and 2 mg/kg, IP) or NSO (21, 42, and 84 μL/kg, IP) for 54 days. Data were expressed as mean ± SEM (*n* = 4). Statistical analysis was performed by one‐way ANOVA and Tukey–Kramer test. ^####^ *p* < 0.0001, ^###^ *p* < 0.001, ^##^ *p* < 0.01, and ^#^ *p* < 0.05 compared to the control group; **p < 0.01, and *p < 0.05 compared to the BPA group. BPA, bisphenol A; KCl, potassium chloride; NSO, *Nigella sativa* oil; PE, phenylephrine; TQ, thymoquinone.

Our results showed that BPA treatment significantly decreased the vasoconstrictive responses to KCl at the concentration of 80 mM in rat aorta rings compared to the control group (*p* < 0.05; Figure 5a,b). Administration of BPA with NSO 21 and 42 μL/kg had a significant effect on the vasoconstrictive responses to KCl at the concentration of 80 mM in comparison with the BPA (*p* < 0.05; Figure 5b). Treatment with TQ alone indicates a considerable decrease in the contractile responses to KCl at the concentrations of 40 mM (*p* < 0.0001) and 80 mM (*p* < 0.05) when compared to the control group. After NSO administration alone, the contractile response to KCl at concentrations of 20 (*p* < 0.05) and 80 mM (*p* < 0.01) was profoundly decreased compared to the control group (Figure 5b).

As shown in Figure 5c,d, BPA showed a significant decrease in the contractile response of rings to PE (10^−7^ M: *p* < 0.05, 10^−6^ M: *p* < 0.0001, 10^−5^ M: *p* < 0.01, and 10^−4^ M: *p* < 0.0001) in comparison with the control. Administration of NSO 21 μL/kg with BPA had a significant effect on the vasoconstrictive responses to PE at the concentrations of 10^−5^ M and 10^−4^ M in comparison with the BPA group (*p* < 0.01). Besides, NSO at the dose of 42 μL/kg along with BPA significantly decreased the vasoconstrictive responses to PE (10^−5^ M: *p* < 0.01 and 10^−4^ M: *p* < 0.05). TQ administration alone showed a significant decrease in the contractile responses to PE (10^−6^ M: *p* < 0.001 and 10^−4^ M: *p* < 0.01; Figure 5c) compared to the control. NSO treatment alone induced a significant decrease in the contractile responses to PE (10^−7^ M: *p* < 0.01, 10^−6^ M: *p* < 0.0001, 10^−5^ M: *p* < 0.0001, and 10^−4^ M: *p* < 0.0001; Figure 5d) as compared with the control group.

### 3.6 Aorta Vasodilation Responses to ACh and SNP

**FIGURE 6:**
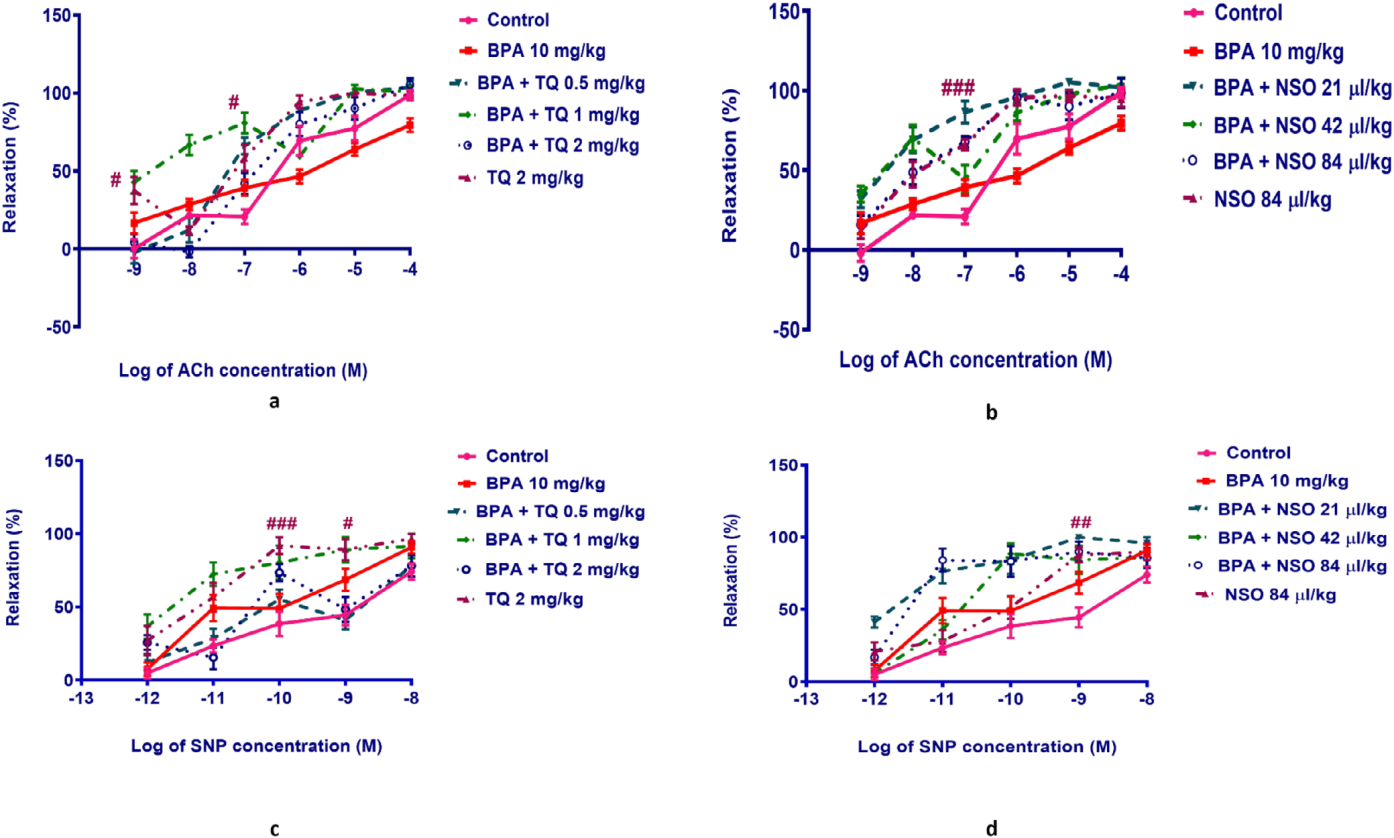
Concentration‐response curve presenting the relaxation responses of PE‐pre‐contracted (10^−6^ M) rat aorta rings to the cumulative concentrations of ACh (a, b) and SNP (c, d). Male Wistar rats were treated with BPA (10 mg/kg, gavage) and/or TQ (0.5, 1, and 2 mg/kg, IP) or NSO (21, 42, and 84 μL/kg, IP) for 54 days. Data were expressed as mean ± SEM (*n* = 4). Statistical analysis was performed by one‐way ANOVA and Tukey–Kramer test. ^###^ *p* < 0.001, ^##^ *p* < 0.01, and ^#^ *p* < 0.05 compared to the control group. ACh, acetylcholine; BPA, bisphenol A; NSO, *Nigella sativa* oil; SNP, sodium nitroprusside; TQ, thymoquinone.

Treatment with TQ at concentrations of 10^−9^ and 10^−7^ M (*p* < 0.05) or NSO at a concentration of 10^−7^ M (*p* < 0.001) alone indicates a significant increase in vasodilatory responses to ACh when compared to the control group (Figure 6a,b). As shown in Table 1, subchronic administration of TQ (*p* < 0.05) or NSO (*p* < 0.001) alone significantly increased the pD_2_ (−log EC_50_) value in comparison with the control group (Table 1).

**TABLE 1:** Effect of TQ, NSO, and BPA on pD_2_ (−log EC_50_) value of acetylcholine in isolated rat aorta.

Groups	pD_2_
Control	6.37 (95% CI: 6.04–6.71)
TQ 2 mg/kg	7.28 (95% CI: 6.91–7.65)^#^
NSO 84 μL/kg	7.72 (95% CI: 7.47–7.96)^###^
BPA 10 mg/kg	6.12 (95% CI: 5.75–6.49)
BPA 10 mg/kg + TQ 0.5 mg/kg	7.24 (95% CI: 7.06–7.42)
BPA 10 mg/kg + TQ 1 mg/kg	8.73 (95% CI: 8.08–9.37)
BPA 10 mg/kg + TQ 2 mg/kg	6.75 (95% CI: 6.51–6.98)
BPA 10 mg/kg + NSO 21 μL/kg	8.47 (95% CI: 8.26–8.69)
BPA 10 mg/kg + NSO 42 μL/kg	8.02 (95% CI: 7.33–8.71)
BPA 10 mg/kg + NSO 84 μL/kg	7.77 (95% CI: 7.45–8.09)

*Note:* Data are presented as mean ± SEM.

Abbreviations: BPA, bisphenol A; CI, confidence interval; NSO, *Nigella sativa* oil; pD_2_, −log EC_50_; TQ, thymoquinone.

^#^ *p* < 0.05 and ^###^ *p* < 0.001 versus control group.

As shown in Figure 6c,d, administration of TQ at 10^−10^ M: *p* < 0.001 and 10^−9^ M: *p* < 0.05 or NSO at 10^−9^ M: *p* < 0.01 alone induced a significant increase in vasorelaxation responses to SNP (Figure 6c,d) compared with the control group.

## 4 Discussion

The beneficial effects of *N. sativa* and its constituent, thymoquinone, in cardiovascular diseases have been demonstrated in previous research works (Danaei et al. 2019; Randhawa et al. 2013; Razavi and Hosseinzadeh 2014; Xiao et al. 2018). Few studies, however, have investigated whether *N. sativa* and TQ can be helpful against BPA‐induced vascular toxicity. In the present study, TQ and NSO effects on BPA vascular toxicity were estimated in HUVECs by measuring cell viability, ROS and NO levels, and the protein levels of p‐eNOS (ser 1177), VCAM‐1, and E‐selectin. Moreover, in another set of our experiments, TQ, NSO, and BPA‐induced alterations on rat aorta MDA level, aortic vasoconstrictor responses to KCl and PE, and vasodilatory responses to ACh and SNP were evaluated.

In the present in vitro study, BPA decreased cell viability and increased ROS and NO formation. Moreover, BPA treatment increased p‐eNOS and VCAM‐1 protein levels. BPA‐induced cytotoxicity and the increase in p‐eNOS protein levels were ameliorated by the pretreatment of HUVEC cells with TQ. Our in vivo study showed that BPA exposure increased the MDA level of aortic tissue and decreased vasoconstriction to KCl and PE. Co‐treatment of TQ or NSO with BPA attenuated MDA levels and also had significant responses in vasoconstriction.

Malondialdehyde (MDA) is considered an important biomarker of oxidative stress produced in lipid peroxidation of tissues (Vahdati Hassani et al. 2017). In the present study, BPA induced a significant increase in MDA levels of aortic tissue. Our results were consistent with previous studies that BPA remarkably increased MDA content in rat aorta (Mohsenzadeh, Razavi, Imenshahidi, Mohajeri, et al. 2021; Rameshrad et al. 2018). Both TQ and NSO significantly reduced BPA‐induced lipid peroxidation in the aorta. However, based on the reduction in MDA levels, NSO at higher doses showed an effect comparable to TQ at its highest dose. This suggests that both agents exhibit strong antioxidant properties, with NSO potentially providing additional protective constituents beyond TQ. Nevertheless, the study did not aim to directly compare their relative efficacy; therefore, the results should not be interpreted as indicating the superiority of one agent over the other. Previous studies have also supported the protective effects of TQ against deleterious effects induced by oxidative stress. For example, TQ can protect from pyrogallol‐induced oxidative stress in rabbit aorta (El‐Agamy and Nader 2012). Moreover, TQ has been shown to reduce serum MDA levels in rabbits thereby diminishing the risk of atherosclerosis (Nader et al. 2010). These findings suggest that the beneficial effects of TQ and NSO might result from their antioxidant capacity.

Oxidative stress results from the imbalance between ROS generation and antioxidant defenses. Oxidative stress can promote nitrosative stress caused by reactive nitrogen species (Zhang et al. 2016). Several studies reported that BPA exposure has been linked to increasing oxidative and nitrosative stress in human endothelial cells (Andersson and Brittebo 2012; Cho et al. 2018). In the current study, BPA significantly increased intracellular ROS and NO levels in HUVECs. However, pretreatment of HUVECs with NSO or TQ at three doses reversed the increased ROS and NO production. Consistent with our results, it has been reported that *N. sativa* and TQ can protect PC12 cells against cytotoxicity induced by serum/glucose deprivation (SGD) (Babazadeh et al. 2012; Mousavi et al. 2010). Our experimental findings suggest that NSO and its active component TQ can protect the HUVECs against BPA‐induced cytotoxicity through an antioxidant mechanism.

In vascular endothelium, NO is synthesized from l‐arginine in the presence of endothelial NO synthase (eNOS). Activation of eNOS requires its essential cofactor tetrahydrobiopterin (BH_4_) for NO production. Under conditions of oxidative stress, BH_4_ is depleted. Reduced levels of BH_4_ uncouple eNOS, leading to superoxide $\left(O_{2}^{-}\right)$ production. The reaction between $O_{2}^{-}$ and NO produces peroxynitrite (ONOO^−^). Both $O_{2}^{-}$ produced by eNOS uncoupling and inactivation of NO by $O_{2}^{-}$ lead to impaired vasorelaxation and hence can cause inflammation and vascular endothelial dysfunction (Chen et al. 2008; Förstermann 2010). In this study, BPA increased ROS levels in HUVECs. In response to oxidative stress, phosphorylation of eNOS at ser 1177 was increased to produce NO and prevent endothelial dysfunction. Endothelial NOS is an important component of vascular homeostasis. Consistent with our findings, activation of eNOS ser‐1177 following oxidative stress has also been reported in previous studies, which this response may indicate an attempt by endothelial cells to maintain NO bioactivity under conditions of oxidative stress (Andersson and Brittebo 2012; Thomas et al. 2002). During inflammation, blood vessels express inducible NO synthase (iNOS) as well as eNOS. Local expression of iNOS as an inflammatory mediator may alter vascular function and contribute to vascular dysfunction (Gunnett et al. 2005). Evaluation of iNOS protein levels can be pursued in future research work to complete the mechanisms of BPA toxicity in the vascular system.

When vascular endothelium is exposed to ROS and oxidative stress, it will activate adhesion molecules including VCAM‐1. The expression of VCAM‐1 on the endothelium and leukocytes has the main role in vascular endothelial dysfunction and possesses predisposing effects on atherosclerosis (Cook‐Mills et al. 2011; Galkina and Ley 2007). In this study, our findings demonstrated that BPA increases VCAM‐1 protein levels in HUVECs. As shown in previous studies, elevated levels of VCAM‐1 protein have been reported after BPA exposure in human endothelial cells (Mohsenzadeh, Razavi, Imenshahidi, Mohajeri, et al. 2021; Rameshrad et al. 2018) and they are in accordance with our result. Pretreatment with NSO or TQ was able to decrease VCAM‐1 protein levels. In other words, NSO and TQ could be candidates for controlling vascular endothelial dysfunction induced by BPA by reducing VCAM‐1 protein levels. These findings are supported by other studies that found *N. sativa* (Abbasnezhad et al. 2019) and TQ (Umar et al. 2015) inhibit VCAM‐1 expression.

In the present study, the E‐selectin protein level showed an increasing trend in the BPA group, but it was not significant. This result is consistent with another research work that showed there is no significant effect on E‐selectin protein levels between BPA and control groups (Rameshrad et al. 2018). E‐selectin is another biomarker of endothelial dysfunction that reflects cellular inflammatory status. Maximal release of E‐selectin was reported 6–12 h after activation of HUVECs and returned to baseline 24 h after activation (Leeuwenberg et al. 1992; Mathew et al. 2010). In this study, BPA increased the E‐selectin protein level, but its effect was not statistically significant. This may be due to the time‐dependent expression of E‐selectin (Leeuwenberg et al. 1992). Therefore, E‐selectin level appears to be detected up to 24 h after activation of HUVECs.

In isolated rat aorta, our results showed that BPA attenuated the contractile responses to KCl (80 mM) and PE (10^−7^–10^−4^ M). PE is an alpha‐1 adrenergic agonist that induces vasoconstriction by releasing Ca^2+^ from the sarcoplasmic reticulum and stimulating receptor‐operative Ca^2+^ channels (ROCCs). Moreover, high extracellular K^+^ ions depolarize vascular smooth muscle cells, increase the influx of extracellular Ca^2+^ through voltage‐operative Ca^2+^ channels (VOCCs), and subsequently lead to vasoconstriction (Rameshrad et al. 2016). An increase in intracellular calcium seems to trigger depolarization and contraction of cardiovascular cells. In this study, the decrease in the contraction of aortic rings to KCl and PE could be related to the disruption of calcium homeostasis by BPA. These results are consistent with previous studies that showed the negative effects of BPA on the intracellular Ca^2+^ concentration (Deutschmann et al. 2013; Liang et al. 2014; Rameshrad et al. 2018).

The present data showed that when NSO was administrated with BPA, a significant effect on vascular responses to KCl and PE was observed. Administration of TQ and NSO alone reduced the contractile response to KCl and PE in comparison with the control. Consistent with our result, TQ has been demonstrated to decrease the tension of the pulmonary artery of rats precontracted by PE. It was suggested that the vasorelaxant effects of TQ can be related to the activation of K_ATP_ channels and the non‐competitive inhibition of serotonin, α1, and endothelin receptors (Suddek 2010). Furthermore, NSO caused an endothelium‐independent inhibitory effect on PE and KCl‐induced contraction. This vasorelaxation effect was mediated by inhibiting Ca^2+^ and K_ATP_ channels and intracellular calcium release (Niazmand et al. 2014). Similar to these results, our study indicated that TQ and NSO decreased the contractile response of aorta rings to PE and KCl. Although we did not directly measure these pathways in the present study, previous reports indicate that TQ and NSO may mediate vasorelaxation via activation of ATP‐sensitive K^+^ channels, serotonin‐ and endothelin‐mediated receptor blockade, intracellular Ca^2+^release inhibition, or modulation of endothelial NO signaling (Darakhshan et al. 2015; Niazmand et al. 2014; Suddek 2010). These mechanisms should be tested directly in future studies using selective pharmacological inhibitors.

ACh induces NO release from the intact endothelial layer and causes vasodilation through muscarinic receptors (endothelium‐dependent vasodilator). In contrast, SNP is an endothelium‐independent agent that induces vasorelaxation through NO production (Rameshrad et al. 2018). We found that BPA showed nonsignificant effects on vasodilatory responses to ACh. Our findings were consistent with previous studies indicating that low to moderate BPA doses or shorter exposure periods may produce only subtle or even absent changes in vascular endothelial function in healthy animals, with more pronounced effects observed at higher doses, longer exposures, or in disease models such as hyperlipidemia or hypertension (Fang et al. 2014, 2015; Feiteiro et al. 2018; Nagarajan et al. 2021).

The observed enhancement of pD₂ values in groups treated with TQ (2 mg/kg) or NSO (84 μL/kg) alone reflects their intrinsic vasorelaxant and endothelium‐protective properties, which are well documented. TQ and NSO improve NO bioavailability, reduce oxidative stress, and modulate Ca^2+^ and K^+^channels, which can lead to enhanced endothelium‐dependent relaxation even in normal aorta. In diabetic and oxidative stress models, extracts from *Nigella sativa* improve ACh‐mediated relaxation. Moreover, TQ protects against endothelial dysfunction induced by oxidative stress ex vivo and acts as a smooth muscle relaxant (El‐Agamy and Nader 2012; Ghayur et al. 2012; Niazmand et al. 2014). Therefore, these higher values reflect more beneficial pharmacology than assay noise.

BPA produced only a mild, non‐significant change in endothelial‐dependent vasodilation in our conditions, while NSO and TQ, which have vasorelaxant and antioxidant effects, increased ACh sensitivity in intact aorta. The higher pD2 in the NSO and TQ groups alone therefore reflects beneficial pharmacology and not assay noise. The direction and magnitude of these effects are consistent with previous reports of vasoprotection by NSO and TQ.

In the present study, histopathological evaluation of aortic tissue using H&E staining did not reveal significant changes. This may be attributed to the relatively low BPA dose, shorter exposure period, the limited sensitivity of H&E staining, and the use of healthy rats instead of hyperlipidemic models (Fang et al. 2014, 2015). These factors will be addressed in future studies to enable more sensitive detection of early atherosclerotic changes.

## 5 Conclusion

In this study, we demonstrated that both NSO and TQ could effectively ameliorate BPA‐induced oxidative stress and vascular endothelial dysfunction. Although both treatments were effective, NSO generally has slightly more vasoprotective effects than TQ, possibly due to its complex phytochemical composition, which may have additive or synergistic effects. TQ, the bioactive component of NSO, provides a more targeted approach for mechanistic studies or potential standardization. Together, our findings suggest that both NSO and TQ are promising candidates for mitigating BPA‐induced vascular injury.

## Author Contributions

Masoumeh Fadishei: investigation, data curation, formal analysis. Mahdieh Sadat Mohsenzadeh: writing – original draft preparation. Bibi Marjan Razavi: conceptualization, formal analysis, methodology, writing – review and editing, supervision. Mohsen Imenshahidi: writing – review and editing. Seyed Ahmad Mohajeri: writing – review and editing. Mahboubeh Ghasemzadeh Rahbardar: writing – review and editing. Hossein Hosseinzadeh: conceptualization, formal analysis, methodology, writing – review and editing, project administration, resources, supervision, funding acquisition. All data were generated in‐house, and no paper mill was used. All authors agree to be accountable for all aspects of work ensuring integrity and accuracy.

## Funding

This work was supported by Mashhad University of Medical Sciences (Grant no. 941399).

## Conflicts of Interest

The authors declare no conflicts of interest.

## Data Availability

The datasets generated and/or analyzed during the current study are available from the corresponding author upon reasonable request.
